# Clinical Management and Outcomes of Dengue Fever and Enteric Fever

**DOI:** 10.7759/cureus.82944

**Published:** 2025-04-24

**Authors:** Qari Muhammad Younas Aman, Maaz Rasul, Amna Khan, Basit Ali, Muhammad Faheem, Aiman Balouch, Imran Qadar Khattak

**Affiliations:** 1 Medicine and Surgery, Lady Reading Hospital, Peshawar, PAK; 2 Medicine and Surgery, Gomal Medical College, DI Khan, PAK; 3 Medicine and Surgery, North West School of Medicine, Khyber Medical University, Nowshera, PAK; 4 Medicine and Surgery, Intern Northwest General Hospital, Peshawar, PAK; 5 Internal Medicine, Combined Military Hospital (CMH) Lahore Medical College and Institute of Dentistry, Lahore, PAK; 6 Internal Medicine, Shaikh Zayed Hospital, Lahore, PAK; 7 Acute Medicine, Scunthorpe General Hospital, North Lincolnshire and Goole NHS Trust, Scunthorpe, GBR; 8 Internal Medicine, Hayatabad Medical Complex, Peshawar, PAK

**Keywords:** clinical guidelines, dengue fever, enteric fever, management protocols, patient outcomes

## Abstract

Background: Dengue fever and enteric fever, two prevalent infectious diseases in tropical and subtropical regions, pose significant public health challenges due to their overlapping clinical manifestations and distinct therapeutic approaches. This study aims to evaluate hospitalization and management protocols for both illnesses, assessing their adherence to clinical guidelines and examining patient outcomes across diverse healthcare settings.

Methodology: A retrospective cross-sectional study was conducted by reviewing hospital records over a five-year period (April 2019-April 2024). A total of 218 individuals diagnosed with enteric fever (n=98) and dengue fever (n=120) were included in the study. Data were retrospectively collected using standardized forms from hospital records, laboratory reports, and discharge summaries to capture demographics, clinical presentations, lab findings, treatments, complications, and outcomes for patients with dengue or enteric fever. Statistical analysis was conducted using SPSS, Version 26 (IBM Corp., Armonk, NY), applying descriptive statistics, chi-square test, t-test, logistic regression, and Cox modeling to evaluate associations between treatment protocols and patient outcomes, with significance set at p<0.05.

Results: Dengue and enteric fever showed distinct clinical patterns: rash and headache were more common in dengue fever, while abdominal pain and diarrhea predominated in enteric fever. Although demographic differences in age, sex, and residence were observed, they were not statistically significant. Improved outcomes in dengue fever were significantly associated with fluid replacement (n=110, 91.67%, p=0.02), reflecting the collective effect of standard supportive care measures. In contrast, antibiotic therapy (n=98, 100.00%, p=0.01) was central to favorable outcomes in enteric fever. Dengue fever was primarily diagnosed through serological testing (n=115, 95.83%), while enteric fever relied on blood cultures (n=78, 79.59%) (χ²=126.98, p<0.0001, OR=0.02). Hospitalization durations were significantly longer in enteric fever, patients staying ≥5 days compared to dengue fever patients (χ²=8.76, p=0.0031, OR=0.39). Recovery without complications was slightly more frequent in dengue fever (n=112, 93.33%) than in enteric fever (n=88, 89.80%), though this difference was not statistically significant (χ²=0.49, p=0.483, OR=0.60). These findings apply to general dengue fever cases only; patients with dengue hemorrhagic fever were managed separately due to differing clinical protocols.

Conclusion: This study highlights the necessity of tailored treatment protocols for enteric and dengue fever, emphasizing the importance of strict adherence to established clinical guidelines to optimize patient outcomes, particularly in resource-limited healthcare settings. While appropriate management, such as antibiotic therapy for enteric fever and supportive care for dengue, is well defined, differentiating between these conditions based solely on clinical presentation remains a significant challenge due to overlapping symptoms with other febrile illnesses. This diagnostic ambiguity underscores the urgent need for more robust, accessible, and rapid diagnostic tools. Furthermore, ongoing education and capacity building for healthcare professionals are essential to enhance clinical judgment, ensure early recognition, and improve compliance with evolving evidence-based practices in the management of febrile illnesses.

## Introduction

Global health systems currently grapple with significant challenges arising from the escalating incidence of infectious diseases, notably dengue fever and enteric fever [1]. These illnesses predominantly affect populations residing in tropical and subtropical regions where healthcare access is limited, overcrowding is prevalent, and sanitation conditions are suboptimal. Epidemiological studies have established a strong correlation between these sociocultural factors and the disease burden, highlighting the pressing need for targeted public health interventions to mitigate these outbreaks and enhance disease management efforts [2].

Dengue fever, caused by the dengue virus and transmitted primarily by bites from Aedes mosquitoes, manifests with a spectrum of clinical presentations ranging from mild febrile episodes to severe forms such as dengue hemorrhagic fever (DHF) and dengue shock syndrome [3]. The pathophysiology of dengue involves complex immunological responses and alterations in vascular permeability, necessitating careful clinical management to prevent severe complications [4]. The treatment protocols emphasize supportive care, including fluid replacement and vigilant monitoring, underscoring the importance of early detection and intervention to improve patient outcomes [5,6].

Enteric fever, largely attributed to infection by *Salmonella typhi* and *Salmonella paratyphi*, presents with prolonged fever, abdominal pain, and can lead to severe complications if not promptly treated [7]. The management of enteric fever is typically multifaceted, requiring timely antibiotic therapy alongside supportive care to alleviate symptoms and prevent further complications. However, the variability in clinical presentations and the availability of healthcare resources complicate the implementation of standardized treatment guidelines, which can negatively impact patient outcomes [8,9].

Research indicates that prolonged hospital stays and increased complication rates may stem from inconsistent application of hospitalization criteria, delayed diagnosis, and inadequate treatment protocols [10]. Additionally, resource-constrained settings often face significant barriers, including a lack of skilled personnel and diagnostic equipment, which exacerbate healthcare disparities. These challenges underscore the critical need for a systematic assessment of existing clinical practices to identify gaps in the implementation of treatment protocols, thus optimizing patient care and resource allocation [11,12].

Objectives

The objectives of this study are to:

Evaluate and compare the hospitalization and management protocols for dengue fever and enteric fever across diverse clinical settings, focusing on treatment effectiveness, adherence to guidelines, and patient outcomes.

Assess the impact of specific interventions such as supportive care, fluid replacement in dengue fever, and antibiotic therapy in enteric fever, on complication rates, hospitalization duration, and recovery outcomes.

Investigate diagnostic accuracy and adherence to clinical protocols in both diseases, identifying gaps in timely diagnosis (e.g., blood cultures for enteric fever) and treatment, and providing evidence-based recommendations for improving patient care in resource-limited environments.

## Materials and methods

Study design and setting

This study was designed as a multi-center retrospective cohort analysis, where patients diagnosed with dengue fever or enteric fever were followed retrospectively from hospital admission to discharge or in-hospital mortality. This design allowed for a temporal evaluation of treatment strategies, disease progression, and clinical outcomes.

Data were collected from six major tertiary care hospitals in Pakistan, including Lady Reading Hospital, Peshawar; Northwest General Hospital, Peshawar; Hayatabad Medical Complex, Peshawar; Shaikh Zayed Hospital, Lahore; and CMH Lahore. These hospitals were selected based on predefined criteria, which included their high patient influx and diverse case representation. Additionally, the hospitals had to have complete electronic and/or paper-based medical records from April 2019 to April 2024. The selected hospitals consistently used standardized diagnostic coding systems and reliably documented clinical progression, interventions, and outcomes. Hospitals that lacked essential diagnostic or follow-up data were excluded to ensure consistency across sites and to maintain the validity of cohort-based comparisons. This selection process ensured a representative and methodologically robust sample of patients diagnosed with dengue fever and enteric fever.

The study period spanned five years, from April 2019 to April 2024, during which all eligible patients meeting the inclusion criteria were identified and included in the study. Each patient's clinical course was followed retrospectively until discharge or in-hospital mortality.

Inclusion and exclusion criteria

The study included patients of any age and gender with a confirmed clinical or laboratory diagnosis of dengue fever or enteric fever between April 2019 and April 2024. The diagnosis was confirmed through standard serological testing, such as the Widal test or dengue serology, blood cultures, or clinical documentation in accordance with national guidelines. Only patients with complete medical records, which included admission notes, progress charts, laboratory and imaging reports, treatment plans, and outcome documentation, were considered for the study. Transferred or referred patients were included in the analysis if their medical records were complete and they met the eligibility criteria, as excluding them would have limited the representativeness of the study population. This ensured a more comprehensive analysis, reflecting real-world clinical scenarios.

Patients were excluded if they had co-existing infections unrelated to dengue fever or enteric fever, such as malaria, tuberculosis, or chronic liver or kidney diseases, to avoid confounding variables. Additionally, patients who left against medical advice or whose hospitalization records were incomplete, lacking critical clinical or outcome data, were excluded from the analysis. Cases with no documented follow-up during hospitalization were also excluded, as this would hinder the evaluation of treatment response and complications. These inclusion and exclusion criteria were applied consistently across all participating centers to ensure data accuracy, minimize selection bias, and facilitate a reliable cohort analysis.

Sample size

The sample size was calculated using the formula for a proportion-based sample size. 

$n=\frac{Z^{2} \text{ x } p\text{ x } (1-p)}{E^{2}}$

Where Z=1.96 (for a 95% confidence level), p=0.10 (10% expected prevalence of dengue fever and enteric fever), and E=0.05 (5% margin of error). The expected prevalence was based on previous studies indicating a 9.8% prevalence (207 of 2116 participants) for enteric fever [13] and a 10.5% prevalence (21,151 of 201,269 suspected cases) for dengue fever [14]. These values were chosen to represent the typical disease burden in the study population, though actual regional prevalence rates may vary. After applying the eligibility criteria, 218 patients were included in the final analysis, exceeding the minimum required sample size of 140 patients. The decision to use the non-probability convenience sampling method was made due to practical constraints in the selection of hospitals that could provide complete and reliable patient data.

Data collection

Data were retrospectively collected by trained research personnel using standardized data abstraction forms to ensure uniformity and minimize transcription errors. The patients were grouped according to their treatment protocols and environmental exposure. The cohorts were based on exposure to specific treatment protocols (e.g., fluid replacement, antibiotic therapy, supportive care) and environmental factors (urban vs. rural residents). This exposure-based classification allowed for an analysis of how these variables influenced clinical presentations and patient outcomes. The study explored the impact of treatment protocols and environmental risks on disease progression, enabling a better understanding of the relationship between exposure and clinical response.

Patient information was extracted from hospital information systems, inpatient medical records, diagnostic laboratory reports, and discharge summaries. A non-probability convenience sampling technique was employed, where hospitals with appropriate resources (i.e., complete medical records, standardized diagnostic protocols, and reliable follow-up data) were selected for inclusion in the study.

Transferred or referred patients were included in the analysis if their medical records were complete and they met the eligibility criteria. Excluding these patients would have limited the representativeness of the study population. This inclusion ensured a more comprehensive analysis, reflecting real-world clinical scenarios.

Demographic data, including age, gender, and district of residence, were recorded to analyze patient characteristics. Clinical presentation at the time of admission was documented, capturing symptoms such as fever duration, gastrointestinal disturbances, hemorrhagic manifestations, and vital signs. Laboratory parameters, including complete blood count, liver function tests, Widal test, dengue serology, and blood cultures, were retrieved along with imaging results when applicable.

Management strategies were categorized based on treatment protocols, including the use of antipyretics, antibiotics, intravenous fluids, corticosteroids, and supportive interventions such as platelet transfusions and ICU care. Hospital course details, such as length of stay, need for critical care, development of complications (shock, gastrointestinal bleeding, hepatic dysfunction), and final outcomes (discharge or in-hospital mortality), were systematically recorded. Patients were stratified into cohorts based on their diagnosis (enteric fever or dengue fever) and treatment regimens, allowing for meaningful comparisons in clinical outcomes. The temporal sequence of treatments and patient responses was carefully documented, ensuring the study met the essential criteria for cohort-based research.

To ensure consistency across sites, a standardized data abstraction form was used for all hospitals, and only those variables available and consistently recorded across all centers (demographics, clinical presentation, diagnostics, management, and outcomes) were included in the final analysis.

Statistical analysis

Data were analyzed using IBM SPSS Statistics for Windows, Version 26.0 (IBM Corp., Armonk, NY). Descriptive statistics were employed to summarize the findings, with continuous variables (such as age and hospital stay) reported as means, medians, and standard deviations, while categorical variables (such as gender, diagnosis, and outcome status) were presented as frequencies and percentages.

Comparative analyses were performed to assess relationships between treatment protocols and patient outcomes. The chi-square test was applied for categorical variables, while independent samples t-tests and Mann-Whitney U tests were used for continuous variables based on data distribution. To further explore the predictors of prolonged hospitalization or in-hospital mortality, binary logistic regression was conducted. Additionally, Cox proportional hazards modeling was used where appropriate to evaluate survival trends. Confounding factors, such as age, disease severity at baseline, and type of therapeutic intervention, were adjusted to ensure valid associations. A p-value of <0.05 was considered statistically significant across all analyses.

Bias reduction strategies

Several measures were implemented to minimize selection, information, and analytical biases. Data abstraction was conducted independently by multiple reviewers, followed by cross-verification to reduce human errors and ensure data accuracy. Only complete and well-documented patient records were included, mitigating the risk of missing data bias. The use of standardized data collection forms ensured consistency across all participating hospitals. Furthermore, statistical adjustments for potential confounders helped enhance the validity of treatment-outcome associations. Data collection was independently verified by a second reviewer at each site to minimize discrepancies and ensure uniformity across centers.

Ethical approval

Ethical approval for this study was obtained from the Hospital Research and Ethical Committee of MTI-Hayatabad Medical Complex, Peshawar (Approval No. 1295). Additionally, verbal consent was secured from consultants at the participating hospitals after clearly explaining the study’s purpose. All patient data were anonymized before analysis to ensure confidentiality and prevent unauthorized access. The study strictly adhered to the ethical principles outlined in the Declaration of Helsinki and followed institutional guidelines for research involving patient records.

## Results

The demographic details of patients with enteric (n=98, 44.95%) and dengue fever (n=120, 55.05%) are presented in Table 1. The mean ages were 27.80±10.70 years for enteric fever and 29.40±12.30 years for dengue fever. The majority of patients in both groups were between 18 and 40 years (n=72, 60.00% for dengue fever; n=60, 61.22% for enteric fever). Males predominated in both groups (n=70, 58.33% for dengue fever; n=54, 55.10% for enteric fever). Urban residents accounted for a larger proportion of cases in both groups, with n=85, 70.83% of dengue fever cases and n=65, 66.33% of enteric fever cases. However, no statistically significant differences were found between groups based on age, gender, or residence (p>0.05). The demographic distribution indicates that both diseases predominantly affect young adults, with urban populations being at higher risk, possibly due to exposure to vector-prone and contaminated environments.

**Table 1:** Demographic characteristics of patients (n=218) with dengue fever and enteric fever The p-value <0.05 was significant. χ², Chi-square; CI, confidence interval; n, sample size; OR, odds ratio; SD, standard deviation

Variable		Dengue Fever (n=120)	Enteric Fever (n=98)	χ² value	p-value	OR (95% CI)
Age (years)	<18 years	20 (16.67%)	18 (18.37%)	0.31	0.855	1.18 (0.55–2.54)
	18–40 years	72 (60.00%)	60 (61.22%)			
	>40 years	28 (23.33%)	20 (20.41%)			
	Mean ± SD	29.40 ± 12.30	27.80 ± 10.70	-	-	-
Gender	Male	70 (58.33%)	54 (55.10%)	0.12	0.733	0.88 (0.50–1.54)
	Female	50 (41.67%)	44 (44.90%)			
Residence	Urban	85 (70.83%)	65 (66.33%)	0.32	0.570	0.79 (0.44–1.43)
	Rural	35 (29.17%)	33 (33.67%)			

Table 2 presents a comparative analysis of the clinical symptom profiles of patients diagnosed with dengue fever and enteric fever. It illustrates the frequency of key symptoms, including fever, headache, vomiting, rash, abdominal pain, and diarrhea, expressed as percentages, with 95% confidence intervals (CIs) represented by error bars in the accompanying chart. While fever was universally present in both groups (100%), significant differences emerged in other symptoms. Abdominal pain was reported more frequently in enteric fever cases (56.12%) than in dengue fever cases (33.33%) (χ²=8.18, p=0.002; odds ratio (OR)=2.49, 95% CI: 1.39-4.46). Rash was significantly more prevalent among patients with dengue fever (37.50%) than in those with enteric fever (8.16%) (χ²=25.69, p<0.001; OR=0.15, 95% CI: 0.06-0.36). Diarrhea was also more common in enteric fever (38.78%) than in dengue fever (18.33%) (χ²=11.56, p=0.001; OR=2.85, 95% CI: 1.50-5.41). In contrast, the differences in headache (65.00% vs. 61.22%, p=0.589) and vomiting (41.67% vs. 46.94%, p=0.478) were not statistically significant. These findings emphasize the distinct clinical presentations associated with each disease, rash is more indicative of dengue fever, while abdominal pain and diarrhea are more suggestive of enteric fever, thereby supporting their value in differential diagnosis and tailored clinical management of febrile illnesses.

**Table 2:** Clinical presentations of patients with dengue fever and enteric fever and their statistical comparisons *The p-value <0.05 was significant​​​.​​​​ χ², Chi-square; CI, confidence interval; n: sample size; OR, odds ratio

Clinical Presentations	Dengue Fever (n=120)	Enteric Fever (n=98)	χ² value	p-value	OR (95% CI)
Fever (%)	120 (100.00%)	98 (100.00%)	-	-	-
Abdominal pain (%)	40 (33.33%)	55 (56.12%)	8.18	0.002*	2.49 (1.39–4.46)
Headache (%)	78 (65.00%)	60 (61.22%)	0.59	0.589	0.84 (0.49–1.45)
Rash (%)	45 (37.50%)	8 (8.16%)	25.69	<0.001*	0.15 (0.06–0.36)
Diarrhea (%)	22 (18.33%)	38 (38.78%)	11.56	0.001*	2.85 (1.50–5.41)
Vomiting (%)	50 (41.67%)	46 (46.94%)	0.44	0.478	1.24 (0.71–2.17)

Table 3 summarizes the diagnostic methods, treatment strategies, hospitalization duration, and patient outcomes for enteric fever (n=98) and dengue fever (n=120). Dengue fever was primarily diagnosed using serology (n=115, 95.83%), whereas blood culture (n=78, 79.59%) was the main diagnostic method for enteric fever (χ²=126.98, p<0.0001, OR=0.02). Antibiotic therapy was exclusively used for enteric fever (n=98, 100.00%), whereas fluid replacement was more common in dengue fever (n=110, 91.67%). Hospitalization durations were significantly different, with 79.59% (n=78) of enteric fever cases staying ≥5 days compared to 60.00% (n=72) of dengue fever cases (χ²=8.76, p=0.0031, OR=0.39). The treatment regimens and clinical outcomes presented in this table apply exclusively to general dengue fever cases and do not encompass DHF, which requires a different clinical management protocol. All treatment strategies mentioned are aligned with standard care for uncomplicated dengue fever. Recovery without complications was slightly higher in dengue fever (n=112, 93.33%) than enteric fever (n=88, 89.80%), but the difference was not statistically significant (χ²=0.49, p=0.483, OR=0.60). The data confirm that serological tests are key for dengue fever, while blood cultures remain essential for enteric fever. The higher hospitalization rate in enteric fever reflects its longer treatment duration due to antibiotic therapy.

**Table 3:** Diagnostic methods, treatment regimens, hospitalization duration, and patient outcomes *p-value <0.05 was significant. χ², Chi-square; CI, confidence interval; n, sample size; OR, odds ratio; SD, standard deviation

Variable		Dengue Fever (n=120)	Enteric Fever (n=98)	χ² value	p-value	OR (95% CI)
Diagnostic methods	Serology	115 (95.83%)	20 (20.41%)	126.98	<0.001*	0.02 (0.01–0.05)
	Blood culture	5 (4.17%)	78 (79.59%)			
Treatment regimens	Antibiotics	0 (%)	98 (100.00%)	136.65	<0.001*	0.10 (0.04–0.23)
	Fluid replacement	110 (91.67%)	50 (51.02%)			
	Antipyretics	120 (100.00%)	98 (100.00%)			
	Supportive care	90 (75.00%)	45 (45.92%)			
Hospitalization duration	<5 days	48 (40.00%)	20 (20.41%)	8.76	0.003*	0.39 (0.20–0.74)
	≥5 days	72 (60.00%)	78 (79.59%)			
	Mean ± SD (days)	5.80 ± 2.30	7.50 ± 3.10	-	-	-
Patient outcomes	Recovery without complications	112 (93.33%)	88 (89.80%)	0.49	0.483	0.60 (0.20–1.80)
	Complications	8 (6.67%)	10 (10.20%)			

The associations between treatment strategies and patient outcomes for dengue fever (n=120) and enteric fever (n=98) are presented in Table 4. Among dengue fever patients, fluid replacement significantly improved recovery rates (n=110, 91.67%, p=0.02, OR=3.21), and supportive care showed a strong positive correlation with recovery (n=90, 75.00%, p<0.001, OR=2.85). For enteric fever, antibiotics led to a 100% recovery rate (n=98, p<0.001), while fluid replacement (n=50, 51.02%, p=0.04, OR=2.01) and supportive care (n=45, 45.92%, p=0.03, OR=1.85) also contributed to favorable outcomes. These results reinforce antibiotics as the primary treatment for enteric fever, while fluid replacement remains a crucial component of dengue fever management, significantly improving patient outcomes. The treatment regimens listed in this table are not mutually exclusive. Patients in both the dengue fever and enteric fever groups may have received multiple treatments simultaneously. While all patients with enteric fever received antibiotics, fluid replacement and supportive care were administered alongside antibiotics in cases of dehydration or other complications. Therefore, the outcomes presented reflect the combined effect of multiple therapeutic interventions.

**Table 4:** Chi-square test results evaluating associations between management protocols and patient outcomes *The p-value <0.05 was significant. χ², Chi-square; CI, confidence interval; n: sample size; OR, odds ratio Note: The treatment regimens are not mutually exclusive, meaning patients could have received more than one treatment simultaneously. For example, all patients with enteric fever received antibiotics, but fluid replacement and supportive care were administered in addition to antibiotics as needed.

Variable	Patient Group	Treatment Protocol	Outcome (%)	χ² value	p-value	OR (95% CI)
Dengue fever	n=120	Fluid replacement	Recovery without complications (91.67%)	5.12	0.02*	3.21 (1.19–8.67)
		Supportive care	Recovery without complications (75.00%)	12.48	<0.001*	2.85 (1.39–5.83)
Enteric fever	n=98	Antibiotics	Recovery without complications (100.00%)	7.89	<0.001*	-
		Fluid replacement	Recovery without complications (51.02%)	4.27	0.04*	2.01 (1.03–3.91)
		Supportive care	Recovery without complications (45.92%)	6.58	0.03*	1.85 (1.05–3.27)

## Discussion

Dengue fever

Dengue fever predominantly affects patients aged 18 to 40 years, with a mean age of 29.40±12.30 years, which is consistent with other studies that suggest this age group is most commonly afflicted by the illness [15,16]. Clinical manifestations in dengue fever typically include fever, rash, and headache, with the rash being prominent in 37.50% of patients. This aligns with previous research that reported a similar clinical profile for patients with dengue, with rash and headache being key distinguishing symptoms [17].

In terms of diagnosis, serological testing was the most common method for confirming dengue cases in our study, which is in line with other studies highlighting the effectiveness of serology in diagnosing dengue fever [18]. This diagnostic method remains crucial for timely detection and intervention, particularly in regions with high dengue prevalence.

When discussing management, supportive care and fluid replacement were significantly correlated with positive outcomes in patients with dengue. In our study, patients who received fluid replacement had a recovery rate of 91.67%, while those who received supportive care had a 75.00% recovery rate. These findings are consistent with earlier research that underscores the importance of fluid management in avoiding severe outcomes in patients with dengue [19]. Dengue fever treatment predominantly focuses on maintaining hydration and managing symptoms rather than using specific antiviral therapies.

Compared to enteric fever, patients with dengue fever had a significantly shorter hospital stay (mean 5.80±2.30 days), which supports findings from other studies emphasizing the quicker recovery time for patients with dengue when treated according to established protocols [17,20,21]. Our study highlights the importance of supportive care and fluid replacement in dengue management, reinforcing the notion that appropriate management can lead to favorable outcomes.

Enteric fever

In contrast, patients with enteric fever, with a mean age of 27.80±10.70 years, were also predominantly within the 18 to 40 age range, as observed in other research [14,15,17]. Enteric fever is characterized by a different set of clinical manifestations, primarily involving gastrointestinal symptoms such as diarrhea, rash, and stomach pain. These symptoms were significantly more common in patients with enteric fever than those with dengue fever, consistent with previous studies that highlighted gastrointestinal distress as key in the presentation of enteric fever [17].

Blood culture was the most common diagnostic tool for enteric fever, supporting earlier findings that emphasize the importance of blood cultures in diagnosing *Salmonella* infections [21,22,23]. This is in contrast to dengue fever, where serology plays a central role in diagnosis. The diagnostic approach for enteric fever underscores the need for more invasive testing, particularly in regions where the disease burden is high.

For treatment, antibiotics were crucial, with all patients with enteric fever (100% recovery rate) receiving antibiotics as part of their treatment regimen. This aligns with established treatment protocols that emphasize the use of antibiotics to treat enteric fever effectively [21,24]. While fluid replacement and supportive care were also administered, the primary focus remains on antibiotic therapy, which directly addresses the bacterial infection causing the illness.

Patients with enteric fever had a longer hospital stay (mean 7.50±3.10 days) compared to patients with dengue fever, which is consistent with research indicating that enteric fever is often associated with more complications, leading to extended hospitalization [25,26]. Our study highlights the importance of early detection and appropriate antibiotic therapy in managing enteric fever and the need for additional interventions in cases with severe complications.

Healthcare access and patient management

Disparities in healthcare access can significantly influence patient management and outcomes for both dengue fever and enteric fever. In regions with limited healthcare infrastructure, timely access to diagnostic tests and appropriate treatment may be delayed, leading to worse outcomes. For dengue fever, lack of access to fluids and supportive care can lead to more severe disease progression. Similarly, for enteric fever, delays in obtaining blood cultures or access to antibiotics can result in prolonged illness and complications. Our study underscores the importance of addressing these disparities to ensure that patients with both dengue fever and enteric fever receive prompt, appropriate care. Our findings emphasize the need for tailored clinical strategies based on the distinct characteristics of dengue fever and enteric fever. By discussing these illnesses separately, we provide a more focused understanding of their clinical, diagnostic, and treatment needs. Furthermore, considering the variations in healthcare access is crucial for optimizing patient outcomes, especially in resource-limited settings. The treatment protocols for each illness must be customized to reflect the unique pathophysiological traits of the diseases, with particular attention to the availability of diagnostic tools and therapeutic options.

Study strengths and limitations

Strengths

This study provides a comprehensive evaluation of the clinical presentations, diagnostic methods, and treatment strategies for enteric fever and dengue fever, offering valuable insights into patient outcomes. One of the study’s key strengths is the large sample size (n=218), which enhances statistical power and reliability, enabling robust comparisons between the two diseases. The retrospective cohort design allowed for the collection of detailed clinical and demographic data, contributing to accurate disease characterization. Validated diagnostic criteria, including blood cultures for enteric fever and serological testing for dengue fever, enhanced diagnostic reliability and minimized misclassification bias. Additionally, the diverse patient population, drawn from both rural and urban healthcare settings across multiple tertiary care hospitals, improves the generalizability of the findings, reflecting real-world disease burden and patient demographics.

To ensure data completeness and accuracy, the study applied stringent inclusion criteria and only included patients with fully documented medical records, eliminating the risk of missing data bias. Multiple data sources, such as electronic health records, inpatient medical charts, and physician notes, were cross-verified to further enhance reliability. Standardized diagnostic and treatment protocols were consistently applied across all participating hospitals, reducing variability in patient management and ensuring comparability between cases. This thorough approach strengthens the robustness of the findings across clinical and demographic subgroups, offering valuable insights into the management of enteric fever and dengue fever in diverse healthcare settings.

Limitations

Despite these strengths, the study has some limitations. As a retrospective study, it focused solely on in-hospital outcomes, without providing long-term follow-up data on post-discharge complications or disease recurrence. This limits the ability to assess longer-term patient prognoses and the impact of treatment strategies beyond hospitalization. The retrospective design inherently carries the risk of bias due to the reliance on existing medical records, which may not capture all relevant patient data or result in inconsistencies in how the data is documented.

The study involved a convenience sample, which may limit the generalizability of the findings to broader populations, especially outside the specific hospitals studied. Although both rural and urban populations were included, differences in healthcare accessibility and referral patterns between these populations may introduce variability in disease presentation and management.

Another limitation is the potential diagnostic challenge in accurately distinguishing between enteric fever and other febrile illnesses, including viral infections such as dengue fever. Misclassification or delayed diagnosis may occur, impacting the accuracy of findings. While validated diagnostic methods such as blood cultures and serological tests were employed, the study was unable to fully address the limitations of diagnostic tests in resource-limited settings, where access to advanced diagnostics may be restricted.

Although multiple data sources were used, the study’s reliance on available medical records could lead to incomplete or inconsistent data, which may affect the overall data reliability. Furthermore, the combined analysis of two distinct diseases, enteric fever and dengue fever, provides a broad understanding of their clinical management, but separating the analytical frameworks for these diseases might have yielded more specific insights.

Suggestions for future research

Future multicenter studies with larger sample sizes, prospective follow-ups, patient monitoring, and assessments of treatment cost-effectiveness could address these gaps and provide valuable insights for healthcare policymakers and clinicians, further strengthening the clinical relevance of these findings.

## Conclusions

This study analyzed the clinical, diagnostic, and treatment profiles of patients with dengue fever and enteric fever, revealing that both diseases predominantly affect young adults, with a higher prevalence among males and urban residents. The clinical symptom profiles of each disease were distinct, with dengue fever showing a higher frequency of rash and headache. In contrast, enteric fever was more commonly associated with abdominal pain and diarrhea. The findings underscore the need for accurate differential diagnosis based on clinical symptoms, as these can guide treatment choices. Diagnostic methods also varied significantly, with serology playing a key role in the diagnosis of dengue fever and blood cultures being essential for enteric fever. Despite these differences, both diseases showed favorable recovery rates when treated according to established protocols, with recovery being slightly more common in patients with dengue fever.

However, it is important to acknowledge that the study's design and the absence of consideration for certain confounding factors, such as comorbidities or variations in healthcare access, may limit the generalizability of the findings. While the association between treatment strategies and outcomes was observed, the study cannot definitively establish causality due to the observational nature of the study. Future research should focus on prospective, multi-center studies that control for confounders to strengthen the conclusions and improve the understanding of disease management. This will also help refine treatment protocols and ensure they are tailored to the local epidemiological context, especially in settings with limited resources.

## Appendices

**Table 5:** Questionnaire for comparative clinical evaluation of patients with dengue fever and enteric fever

Section	Statement	Options
Demographic Information	Patient ID	__________
	Age (in years)	__________
	Categorized	<18 years; 18–40 years; 40 years
	Gender	Male; Female
	Area of Residence	Urban; Rural
Clinical Characteristics	Fever	Present; Absent
	Headache	Present; Absent
	Vomiting	Present; Absent
	Rash	Present; Absent
	Abdominal Pain	Present; Absent
	Diarrhea	Present; Absent
Diagnostic Methods	Diagnostic Method Used	Serology; Widal Test; Blood Culture; CBC/LFTs/Others
Treatment Regimen	Antibiotics Used	Yes; No ( If yes, specify: ____________)
	Fluid Replacement Therapy	Yes; No
	Antipyretics Administered	Yes; No
	Supportive Care Provided	Yes; No ( If yes, specify: ____________)
Hospitalization	Duration of Hospital Stay (in days)	__________ (<5 days, ≥5 days)
	Ward of Admission	General Ward; ICU; Pediatrics; Emergency; Other
Patient Outcome	Outcome at Discharge	Recovery Without Complications, Recovery With Complications, Referred, LAMA, Death
	Complications	Shock; Hemorrhage; Hepatic Dysfunction; Relapse; Other
Follow-Up	Follow-Up Conducted After Discharge	Yes; No
	Mode of Follow-Up	Telephonic; Physical Visit; Other
	Follow-Up Duration	7 Days, 14 Days, 30 Days, Other
	Clinical Status at Follow-Up	Symptom-Free; Mild Symptoms; Readmitted; Deceased; Lost to Follow-Up
	Medication Adherence	Good; Partial; Poor; Not Applicable
	Post-Discharge Investigations	Yes; No ( If yes, specify: ____________)
